# Lattice oxygen self-spillover on reducible oxide supported metal cluster: the water–gas shift reaction on Cu/CeO_2_ catalyst†

**DOI:** 10.1039/d1sc01201k

**Published:** 2021-05-12

**Authors:** Ya-Qiong Su, Guang-Jie Xia, Yanyang Qin, Shujiang Ding, Yang-Gang Wang

**Affiliations:** Department of Chemistry and Guangdong Provincial Key Laboratory of Catalysis, Southern University of Science and Technology Shenzhen Guangdong 518055 China wangyg@sustech.edu.cn; School of Chemistry, Xi'an Key Laboratory of Sustainable Energy Materials Chemistry, MOE Key Laboratory for Nonequilibrium Synthesis and Modulation of Condensed Matter, State Key Laboratory of Electrical Insulation and Power Equipment, Xi'an Jiaotong University Xi'an 710049 China; Laboratory of Inorganic Materials and Catalysis, Schuit Institute of Catalysis, Eindhoven University of Technology P. O. Box 513 5600 MB Eindhoven The Netherlands

## Abstract

In this work we have tackled one of the most challenging problems in nanocatalysis namely understanding the role of reducible oxide supports in metal catalyzed reactions. As a prototypical example, the very well-studied water gas shift reaction catalyzed by CeO_2_ supported Cu nanoclusters is chosen to probe how the reducible oxide support modifies the catalyst structures, catalytically active sites and even the reaction mechanisms. By employing density functional theory calculations in conjunction with a genetic algorithm and *ab initio* molecular dynamics simulations, we have identified an unprecedented spillover of the surface lattice oxygen from the ceria support to the Cu cluster, which is rarely considered previously but may widely exist in oxide supported metal catalysts under realistic conditions. The oxygen spillover causes a highly energetic preference of the monolayered configuration of the supported Cu nanocluster, compared to multilayered configurations. Due to the strong metal–oxide interaction, after the O spillover the monolayered cluster is highly oxidized by transferring electrons to the Ce 4f orbitals. The water–gas-shift reaction is further found to more favorably take place on the supported copper monolayer than the copper-ceria periphery, where the on-site oxygen and the adjacent oxidized Cu sites account for the catalytically active sites, synergistically facilitating the water dissociation and the carboxyl formation. The present work provides mechanistic insights into the strong metal–support interaction and its role in catalytic reactions, which may pave a way towards the rational design of metal–oxide catalysts with promising stability, dispersion and catalytic activity.

## Abstract 2

The lattice oxygen on the reducible CeO_2_ support could self-spillover to surface of Cu cluster, generating the on-site oxygen to promote the catalytic water–gas shift reaction.

## Graphical abstract

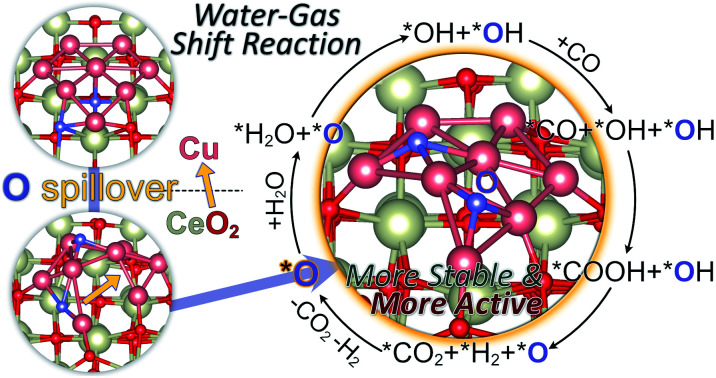

## 1 Introduction

Understanding the role of oxides in catalysis has evolved over time. Initially, oxides were simply used as supports to disperse catalysts for maximum utilization of catalysts. Later, it was gradually realized that oxides can not only regulate the electronic structure and reactivity of the catalyst, but also participate in the reaction and activate the reactants because of the so-called strong metal–support interaction (SMSI).^1–3^ Among oxides, reducible oxides such as TiO_2_ and CeO_2_, have exhibited prominent performance due to their excellent redox properties where metal cations can frequently convert between low and high oxidation states.^4^ Especially, oxygen vacancies are easily available on the surfaces of oxides and can play an important role in the catalytic process: they can be used as either reducing active centers for reactant species^5^ or sites for anchoring the catalytic nanoparticles and single atoms.^6^ Although extensive studies have been carried out on the role of reducible oxides during the catalytic process, controversies still exist in understanding how they behave and therefore modify the structures of the catalyst, active sites and even the reaction mechanisms. In this regard, oxide supported-metal nanocatalysts are among the most important materials in heterogeneous catalysis,^7,8^ and the main challenges are to explore the nature of catalytically active site/area under realistic reaction conditions due to the complexity of metal–support interaction.^9^ The catalyst morphology strongly depends on the synthesis or reaction conditions.^10,11^ Besides, strong metal–support interaction may result in the reconstruction of the metal–support interface, rather than the simple combination of metal particles and supports.^12^ Previously, a large number of studies contributed to the investigation of metal–support interaction,^3,13^ but the resulting local structure change and its influence on catalytic reactivity are still ambiguous. Theory has played an important role in advancing our understanding of catalytic processes on simplistic static models with specific catalytic sites but rarely addresses how they may change under realistic conditions. Recent advances in computational methodologies have allowed us to simulate more complex models of catalyst reactivity and discovered new mechanistic routes that elucidate the principles of reactivity in complex environments. In the current paper, we discuss the influence of SMSI on the active sites and catalytic performance by means of large-scale *ab initio* molecular dynamics simulations, DFT calculations and microkinetic modeling. We choose a prototypical model reaction: the water gas shift reaction on CeO_2_ supported copper clusters, given the abundance of experimental literature and the hotly debated topics on the charge state and the nature of copper active centers.

Cu/CeO_2_ catalysts are highly active for a number of important reactions, such as CO_2_ hydrogenation and water–gas-shift (WGS) conversion, owing to the strong interaction between CeO_2_ and supported Cu particles or nano-clusters (NCs).^14,15^ In the case of low-temperature WGS reactions, it has been generally proposed that the active sites are located at the Cu–CeO_2_ interface.^15,16^ Recently, Shen and co-workers suggested that the Cu^+^ site and the neighboring V_O_–Ce^3+^ site at the interface of Cu–CeO_2_ are the active sites for WGS reactions.^15^ Rodriguez *et al.* proposed that the key to the high catalytic activity of inverse CeO_2_/Cu(111) catalysts for WGS reactions is the nanosize of the ceria particles and the effects of the metal/oxide interface.^17^ Flytzani-Stephanopoulos and co-workers thought that only the strongly bound Cu–[O_*x*_]–Ce species associated with the surface oxygen vacancies of ceria are active for the low-temperature WGS reaction. Meanwhile, Huang and co-workers found that WGS reactions can smoothly occur at the interface of Cu–Cu suboxide by combining experimental and theoretical tools.^18^ Caldas *et al.* also suggested that the active sites of WGS reactions over Cu/CeO_2_ catalysts are distributed at the Cu–Cu_2_O interface rather than the Cu–CeO_2_ periphery.^19^ Ahn *et al.* found that a higher Cu dispersion and a smaller Cu particle size on ceria have an enormous effect on the WGS reactivity in the low-temperature region.^20^ In these arguments, it is generally believed that the active sites of WGS reactions over Cu–CeO_2_ catalysts are formed owing to the SMSI. The SMSI between copper and ceria results into the prevention of Cu sintering,^21^ and highly dispersed Cu monolayers and/or bilayers may be created.^15,22,23^ In previous theoretical studies,^17,24^ the Cu/CeO_2_ system is usually modelled with carefully selected metal clusters on ceria under static conditions, in which their reconstructions at finite temperature are out of consideration. The local geometry of ultra-fine Cu NCs on CeO_2_ under actual experimental conditions and the possible mechanisms of WGS over Cu/CeO_2_ are still obscure.

Herein, to resolve the role of the Cu–CeO_2_ interface in the water gas shift reaction, we systematically investigate the local geometries, electronic properties and catalytic performances of CeO_2_–supported Cu NCs by means of density functional theory (DFT) calculations and *ab initio* molecular dynamics (AIMD). CeO_2_(111) is chosen to mimic the ceria support, as it is one of the stable CeO_2_ facets, and generally used in experimental and theoretical studies. It is demonstrated that Cu NCs prefer to form a monolayer on CeO_2_ and the lattice oxygen spillover dynamically takes place from underside of the Cu monolayer to its top surface, in virtue of strong interaction between the Cu monolayer and CeO_2_. Charge analysis reveals that most Cu atoms are highly positively charged with the lattice oxygen spillover. The reaction mechanism of WGS reaction with the participation of the on-site oxygen and the catalytically active sites is further discussed.

## 2 Computational methods

### 2.1 DFT calculations

We carried out spin-polarized DFT calculations as implemented in the Vienna *ab initio* simulation package (VASP).^25^ The ion–electron interactions were represented by the projector-augmented wave (PAW) method and the electron exchange–correlation by the generalized gradient approximation (GGA) with the Perdew–Burke–Ernzerhof (PBE) exchange–correlation functional.^26^ The Kohn–Sham valence states were expanded in a plane-wave basis set with a cut-off energy of 400 eV. The Ce(5s, 5p, 6s, 4f, 5d), O(2s, 2p), Cu(3d, 4s), C(2s, 2p) and H(1s) electrons were treated as valence states. Grimme's D3 corrections were involved to describe the dispersion interactions.^27^ The DFT + *U* approach was used, in which *U* is a Hubbard-like term describing the on-site coulombic interactions.^28^ This approach improves the description of localized states in ceria, where the standard LDA and GGA functionals fail. For Ce, a value of *U* = 4.5 eV was adopted.^29^

For Cu/CeO_2_(111), we use a periodic ceria slab with a (4 × 4) surface unit cell. For Brillouin zone integration, a 1 × 1 × 1 Monkhorst–Pack mesh was used. The bulk equilibrium lattice constant (5.49 Å) from the previous calculation at the PBE + *U* level (*U* = 4.5 eV) was used. The CeO_2_(111) slab model consists of three Ce–O–Ce layers and a vacuum gap of 15 Å. The atoms in the bottom layer were frozen to their bulk positions and only the top two Ce–O–Ce layers were relaxed. The climbing image nudged-elastic band (CI-NEB) algorithm^30^ was used to identify the transition states in the elementary reaction steps of WGS reactions. The setting for CI-NEB is completely the same as geometry optimization to keep the consistency of computational data. The energy convergence criterion was set to be 10^−6^ eV, and the atomic forces in the optimized structures were smaller than 0.02 eV Å^−1^.

We computed the IR intensity of stretching vibrational modes of adsorbed water (H_2_O and D_2_O). For this purpose, finite difference analysis was employed to those stable CO adsorption structures. The first-order IR intensity of the *i*th mode is given by Porezag *et al.*^31^1
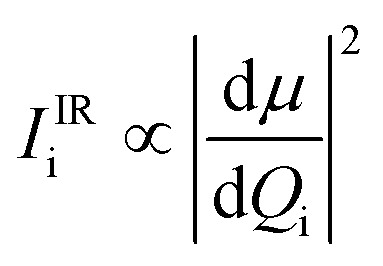
where *Q*_i_ is referred to as a normal mode coordinate and *μ* is the electric dipole moment of the system.

The simulated IR spectra were presented in terms of the Lorentzian expansion of the first-order IR intensity with a linewidth of 10 cm^−1^.

### 2.2 AIMD simulations

In *Ab Initio* Molecular Dynamics (AIMD) simulations, CP2K is used to study the dynamic interactions by using the Quickstep module.^32^ The electron density is expanded in a Gaussians and auxiliary plane wave (GPW) double zeta Gaussian basis set^33^ with an energy cutoff of 400 Ry, and Goedecker–Teter–Hutter (GTH) type pseudopotentials^34^ are used. The PBE functional^26^ with D3 dispersion corrections^27^ is implemented to calculate the total electronic energy and atomic forces. To ensure the AIMD simulations in CP2K is at the same footage of DFT calculations in VASP, the Hubbard-like term of the DFT + *U* approach was tested with the details shown in the ESI.† The AIMD lasts for 15 ps with a time step pf 1.0 fs. To simulate the as-prepared catalyst, the temperature is controlled by using Nosé–Hoover thermostats^35^ at 773 K, which is the typical calcination temperature for catalyst preparation.^36–38^ Although this temperature is slightly higher than the working temperatures for low-temperature WGS catalysts, the estimated rates could still suggest the occurrence of O spillovers with the details shown in the ESI.†

## 3 Results and discussion

### 3.1 Geometry of Cu NCs on CeO_2_(111)

The strong metal–oxide support interaction plays a crucial role in the geometric and electronic properties of catalyst particles and in turn strongly affects the catalytic activity. It is important to understand the preferable structure of Cu particles on cerium oxide before exploring the catalytic process. Therefore, we employed a genetic algorithm in conjunction with the DFT (GA-DFT) method to explore the possible geometries of small Cu NCs on CeO_2_(111). This method allows us to perform a global geometry search by screening more than 500 structures which are randomly generated by arranging the possible positions of atoms on the basis of reasonable bonding distances and DFT calculated energies. During the GA-DFT calculations, both the Cu NCs and the first layer of the ceria substrate are allowed to move to guess the new structures. The detailed information of GA-DFT can be found in the ESI.†

**Fig. 1:**
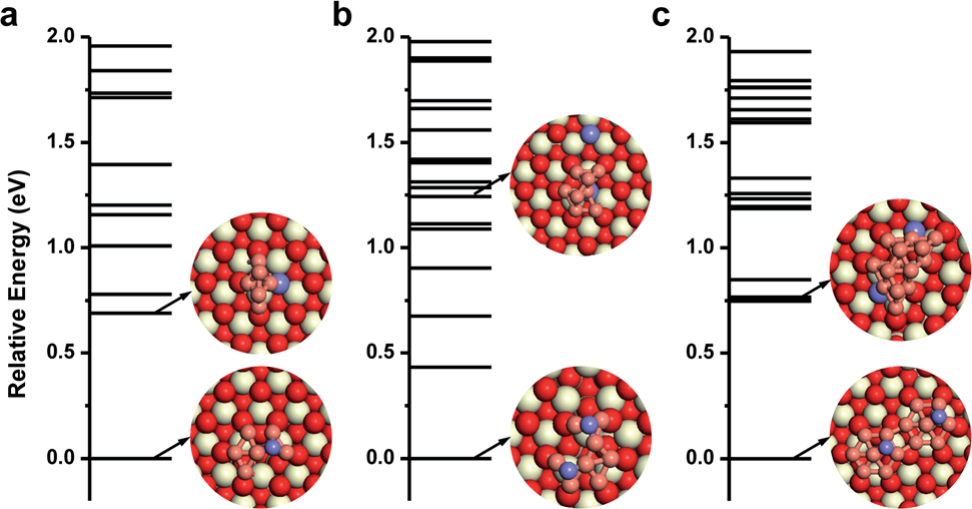
Geometric configurations and relative potential energies of (a) Cu_6_, (b) Cu_8_, and (c) Cu_14_ on CeO_2_(111) obtained by GA-DFT calculations. The red, white and brown balls are O, Ce and Cu atoms respectively, while the spilled-over O atoms are represented in blue.

**Fig. 2:**
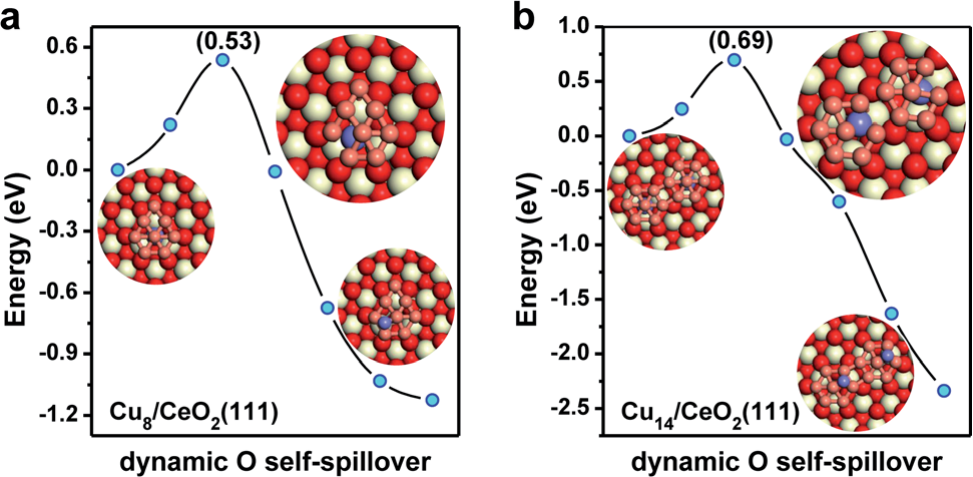
The O spillover process of monolayered Cu_8_ and Cu_14_ on CeO_2_(111) determined by the CI-NEB method. (a) Cu_8_ cluster and (b) Cu_14_ cluster. The colors are the same as those in Fig. 1.

For the single Cu atom, it prefers to adsorb at the oxygen hollow site with an adsorption energy of −2.90 eV as shown in Fig. S1.† Its diffusion barrier on CeO_2_(111) is only 0.89 eV. Considering that the usual experimental temperature for preparing the Cu/CeO_2_ catalyst is as high as 773 K, it is expected that single Cu atoms could agglomerate together to form Cu NCs *via* Ostwald ripening.^39^ For Cu_2_ and Cu_3_ clusters, without enough Cu atoms, there only exist single linear or trigonal planar configurations. The smallest multilayered Cu cluster is Cu_4_, as shown in Fig. S2,† which is more stable than its monolayered configuration by 0.2 eV. However, starting from Cu_5_, although the Cu clusters could adsorb in either a monolayered or a multilayered configuration, the monolayered configuration with oxygen spillover becomes the most stable. Interestingly, when more Cu atoms are involved, the spontaneous spillover of the lattice O from CeO_2_ to Cu is observed, as shown in Fig. 1 and S3.† With one O spillover, the monolayered configuration of the Cu_6_ cluster is considerably more stable than the multilayer one by 0.69 eV. In the case of Cu_7_, it also prefers to adopt a monolayered hexagonal planar configuration with high symmetry, and one lattice oxygen is transferred to its top as shown in Fig. S2.† Analogously, with two O spillover, the monolayered configuration of Cu_8_ on CeO_2_(111) is thermodynamically more stable than the corresponding multilayered configuration even by 1.24 eV. With two hexagons, as shown in Fig. 1, the topological structure of the monolayered Cu_14_ cluster is organized by adjoining the replicas of the monolayered Cu_7_, in which two lattice O on CeO_2_ are spilled over to Cu_14_ (in Fig. S4†). In a word, as shown in Fig. 1, when Cu clusters with more than five Cu atoms are adopted on the CeO_2_(111) surface, the monolayered configurations with O spillover from the oxide surface are thermodynamically more favorable than the multilayered ones without O spillover. These results also suggest that with O spillover the interaction between the monolayered Cu cluster and CeO_2_ can effectively offset the cohesion of multilayered Cu clusters. Besides, according to the stable configurations in Fig. 1, it can be expected that probably the larger the cluster size, the more O spillover could take place, although their quantitative relationship is beyond the scope at the stage. The spillover processes of lattice oxygen beneath the monolayer of Cu NCs are further investigated by CI-NEB calculations. As shown in Fig. 2a, one needs to overcome a barrier of 0.53 eV to spillover one lattice O originally beneath the monolayer of the Cu_8_ cluster to its top, while in the case of Cu_14_, it is 0.69 eV for the spillover of two lattice O. These spillover barriers are much lower than the diffusion barrier of single Cu adatoms on CeO_2_(111) around 0.89 eV. More importantly, the O spillover is a thermodynamically favorable process. Once the spillover of lattice O takes place, the reverse reaction becomes hard. In addition, considering that the subsurface O vacancies are also common on CeO_2_ surfaces, the migration of surface O vacancies, generated from the O spillover, into the subsurface is investigated in Fig. S5,† but due to the metal–support interaction between Cu and CeO_2_, the process is quite endothermic by 1.86 eV.

### 3.2 AIMD simulations of the lattice-oxygen spillover

**Fig. 3:**
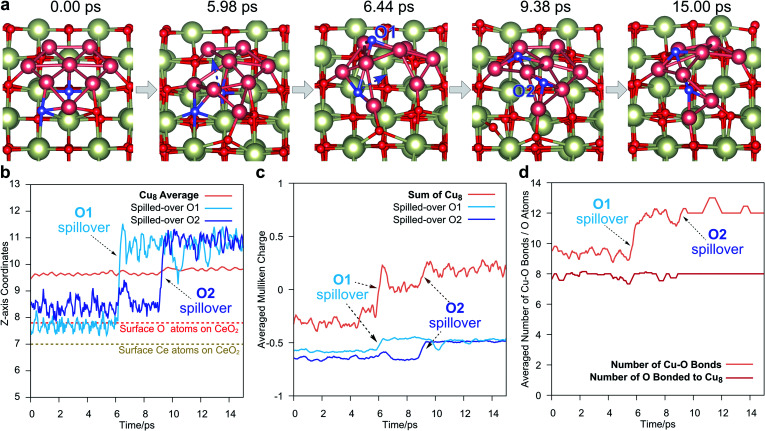
Spillover takes place in the AIMD with the monolayer Cu_8_ cluster configuration on the CeO_2_(111) surface. (a) Typical configurations in AIMD of 15 ps. During the simulation, two O spillovers take place. The colors are the same as those in Fig. 1, and the spilled-over O is colored in blue. (b) The *Z*-axis coordinates of Cu_8_ and the two spilled-over O in AIMD. The label of the spilled-over O is shown in (a). (c) Mulliken charges of Cu_8_ and the two spilled-over O in AIMD. For smoothing the curve, the charges are averaged by the data of the following 0.5 ps. (d) The number of Cu–O bonds (brown line) and the total O atoms bonded to Cu_8_ (red line). The Cu–O bond is expected to be formed within 2.43 Å, which is determined by the radial distribution function (RDF) of O atoms around Cu, as shown in Fig. S6.† The numbers on both curves are also averaged by the data of the following 0.5 ps.

To explore the dynamic behavior of Cu NCs on CeO_2_(111) at the preparing temperature, *ab initio* molecular dynamics (AIMD) simulations were carried out as shown in Fig. 3. We consider a monolayer Cu_8_ cluster supported on a clean CeO_2_(111) surface and perform the simulations at 773 K, corresponding to the usual experimental temperature for preparing the Cu/CeO_2_ catalyst.^40,41^ Without surprise, we indeed observe the spillover of lattice oxygen to the Cu cluster. As shown in Fig. 3a, at around 5.98 ps, the Cu on the left edge of the Cu_8_ monolayer will distort from its original position, which forms a “opening” in the Cu network. Then, the O under the middle Cu atom will soon cross through this “opening” to spillover to the upper side of the Cu monolayer, which can be directly observed from the *Z*-axis coordinates in Fig. 3b. This O will bind with the neighboring three Cu after the spillover, and its Mulliken charge will have a 0.11 |*e*| increase, as shown in Fig. 3c, due to the difference between the electronegativity of Ce and Cu. Because the Cu_8_ is oxidized, the average Mulliken charge of Cu_8_ also increases from ∼−0.29 |*e*| to ∼0.03 |*e*|. These charge analyses clearly demonstrate the Cu monolayer is oxidized by the CeO_2_ support. After the first O spillover, a second spillover is also observed around 9 ps. Similar to the case of the first spillover, the Mulliken charge of O and Cu_8_ both increases by ∼0.16 |*e*| and ∼0.17 |*e*|, respectively.

We also have a statistic on the number of total formed Cu–O bonds and the total number of O bonded to Cu_8_ in the AIMD, as shown in Fig. 3d. The Cu–O bond is expected to be formed within 2.43 Å in the AIMD trajectories, which is determined by the statistic of the averaged radial distribution function (RDF) of O atoms around Cu, as shown in Fig. S6.† As shown in Fig. 3d, during the O spillover process, the total number of Cu–O bonds (brown line) increases, but the number of O atoms appearing in these Cu–O bonds (red line) remains fairly a constant. This implies that only the O originally bond to Cu might have such spillover from the CeO_2_ support to the Cu cluster, which accords with the result of DFT calculation in Fig. 2. Meanwhile, other crystal O atoms on the CeO_2_ could not migrate to Cu, at least in our AIMD time scale. In addition, what should be noticed is that Cu_8_ is a very small model here. For a larger cluster in experiments, according to our AIMD study, such O spillover may take place at the edge of the Cu cluster with the distortion of Cu atoms at the interface between Cu and CeO_2_.

### 3.3 Electronic structure of Cu–CeO_2_

**Fig. 4:**
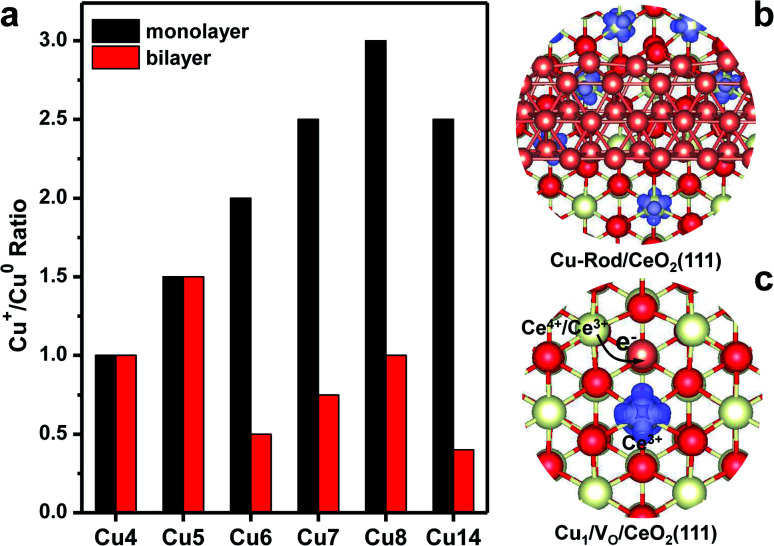
Periphery of Cu–CeO_2_. (a) Cu^+^/Cu^0^ ratio in the Cu NCs supported on CeO_2_(111). (b) The calculated iso-surfaces of spin density at the interface between the bilayer Cu-rod and CeO_2_(111). (c) The calculated iso-surfaces of spin density of Cu adatoms on the oxygen vacancies of the CeO_2_(111) surface. The red, white and brown balls are O, Ce and Cu atoms respectively.

The local electronic structures at the Cu–CeO_2_ periphery play a critical role in several catalytic reactions, such as WGS conversion, CO_2_ hydrogenation and CO oxidation. Recently, Shen and co-workers suggested that the Cu^+^ site and the neighbouring V_O_–Ce^3+^ site at the interface of Cu–CeO_2_ are the active sites for WGS reactions.^15^ In this study, we have found that the strong copper–ceria interaction brings about electron transfer from copper to ceria. As a result, some Cu atoms are in the Cu^+^ oxidation state, and the excess electrons are localized into the 4f-orbital of Ce atoms to produce Ce^3+^ cations. The number of Cu^+^ cations in the supported Cu NCs strongly depends on their morphologies. We determine the number of Cu^+^ cations by counting the total numbers of Ce^3+^ cations in the system, and this method was generally used in previous studies experimentally and theoretically.^42–44^Fig. 4a shows the Cu^+^/Cu^0^ ratio in one Cu NC supported on CeO_2_(111). For bilayer Cu NCs, the Cu^+^/Cu^0^ ratio generally is under 1.0, except for Cu_5_. With the increasing size of bilayer Cu NCs, the Cu^+^/Cu^0^ ratio is evidently declining. For bilayer Cu_14_, the Cu^+^/Cu^0^ ratio is merely 0.4. We also considered a larger bilayer Cu-rod shown in Fig. 4b, in which the Cu^+^/Cu^0^ ratio decreases to 0.24. Fig. 4b shows the excess electron (Ce^3+^) distribution at the interface between the Cu-rod and CeO_2_(111), which distinctly reflects the Cu^+^/Cu^0^ ratio. Meanwhile, the Cu^+^/Cu^0^ ratio in monolayer Cu NCs generally is above 1.5, and even as high as 3.0 for Cu_8_. The results manifest that most Cu atoms in the Cu monolayer are oxidized to Cu^+^ while remaining Cu^0^ in the Cu bilayer.

We further investigated the influence of oxygen vacancies on the Cu^+^/Cu^0^ ratio in Cu NCs. With the formation of one V_O_ on the CeO_2_(111) surface, two Ce^3+^ cations are produced neighboring V_O_. When one Cu atom is bound to V_O_, one excess electron is transferred into the 4s-orbital of this Cu atom from one of these two Ce^3+^ cations. Consequently, one Ce^4+^ cation is reproduced, and the Cu adatom accommodates one excess electron in a negative charge state. Fig. 4c shows the spin-density of the Ce^3+^ cation neighboring the Cu adatom on V_O_. Density of states (DOS) analysis further verifies that one excess electron is filled into the 4s-orbital of the Cu adatom (Fig. S7†). When one oxygen was removed from the periphery of Cu–CeO_2_, we found that the number of excess electrons transfer from copper is reduced. As a result, we expect that the Cu^+^/Cu^0^ ratio gets lowered. These results unravel that the existence of oxygen vacancies at the periphery of Cu–CeO_2_ sacrifices the number of Cu^+^ cations due to electron back-donation, in agreement with previous observations.^45,46^ We mention that we also performed Bader charge analysis for Cu clusters on CeO_2_ as shown in Table S1.† The total charges of Cu clusters also represent a similar trend to the number of Ce^3+^ for the monolayer Cu clusters. Most Cu atoms in the Cu monolayer are in the oxidized state though it is indeed not easy to assign an accurate oxidation number for each Cu atom.

Recently, Shen and co-workers observed ultra-fine dispersed Cu bilayers and monolayers on a CeO_2_ support by scanning transmission electron microscopy (STEM).^15^ In their catalyst, which is synthesized by hydrogen reduction of a CuO/CeO_2_ precursor, the observed Cu^+^/Cu^0^ ratio is 1.22. With the increase of hydrogen reduction temperatures, the Cu^+^/Cu^0^ ratio sharply decreases due to the formation of oxygen vacancies. That experimental observation accords well with our theoretical findings. However, the quite high Cu^+^/Cu^0^ ratio indicates that the dominant Cu NCs on CeO_2_(111) are in the form of atomic monolayers. In their observation, the Cu catalyst mainly contains plate-shaped copper clusters with an average width of circa 1.0 nm, which agrees well with our developed Cu_14_ monolayer with a width of circa 1.1 nm. Meanwhile, Zhu's group found that the charge transferred per Cu atom is around 1.02 at a copper coverage of 0.22 ML on ceria by titration of Ce^3+^, and the supported copper is in the form of the Cu monolayer with an average height of 0.22 nm.^42^ Therefore, we suggest that the small Cu NCs on CeO_2_ facets prefer to be in the form of monolayers with a promising stability due to SMSI between copper and ceria.

### 3.4 Mechanistic nature for the WGS reaction

**Fig. 5:**
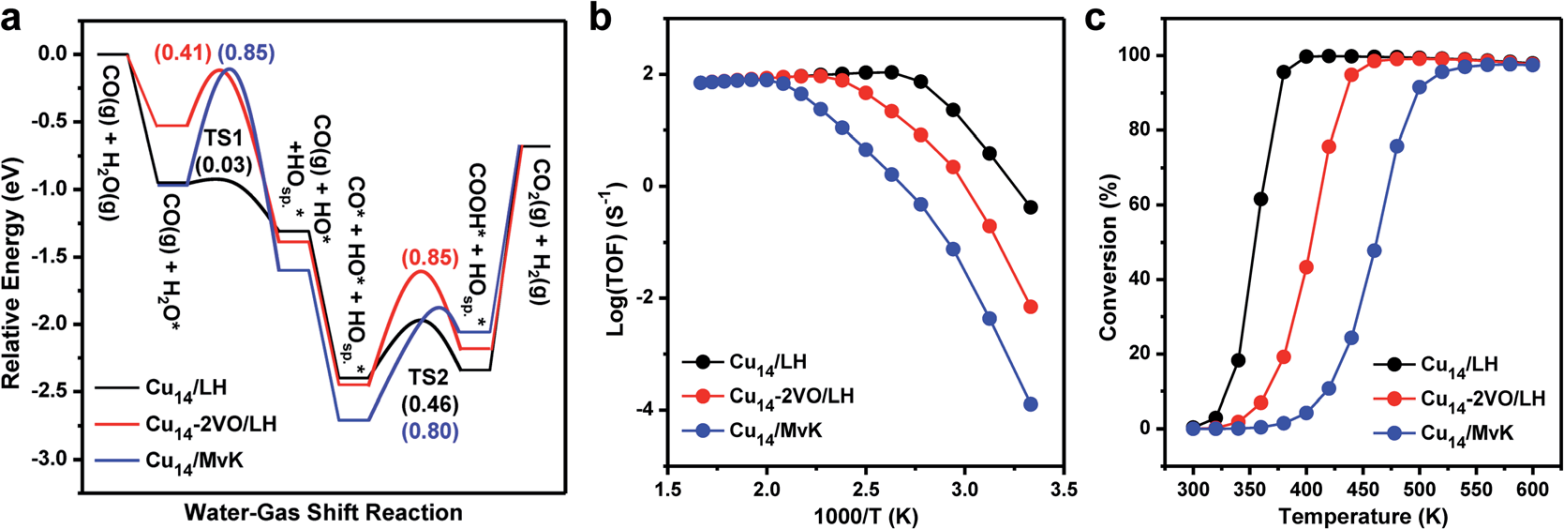
WGS reactions over the Cu/CeO_2_ interface *via* LH and MvK mechanisms. (a) Potential energy diagrams of WGS reactions by monolayered Cu_14_/CeO_2_(111) with two O spillover and Cu_14_–2VO/CeO_2_(111) with the removal of two O spillover, respectively. The O_sp._ represents the spilled-over O up on the Cu cluster. (b) The corresponding Arrhenius plots of the rate of WGS reactions. (c) The corresponding WGS conversions.

We further investigated the reaction mechanisms of WGS on the Cu NCs/CeO_2_ surface. We employed a Cu_14_ monolayer with two O spillover (Cu14-a) as a model to explore the WGS reaction by Cu–CeO_2_. Firstly, we considered the WGS reaction *via* the Langmuir–Hinshelwood (LH) mechanism over Cu_14_/CeO_2_(111) (Fig. S8 and Table S2†), and Fig. 5a shows the corresponding energy profile. The LH mechanism is considered as the following steps: (i) a water molecule adsorbs on the supported copper clusters and dissociates into hydrogen and hydroxyl intermediates (*i.e.* *H on O_sp._ and *OH); (ii) a CO molecule adsorbs on the supported copper clusters and then combines with the *OH, forming a carboxyl intermediate (*COOH); (iii) the carboxyl intermediate dehydrogenates and two hydrogen intermediates combine with each other, completing the catalytic cycle. The water adsorption energy is −0.95 eV, and the following dissociation needs to overcome an energy barrier of merely 0.03 eV. The subsequent CO adsorption is moderate (*E*_ads_ = −1.09 eV), and the COOH formation requires an energy barrier of 0.46 eV.

To investigate the influence of the oxidation state of Cu atoms, we removed these two spilled-over O atoms, and named the model Cu_14_–2V_O_ (Fig. S9 and Table S2†). Following the same pathway, the water adsorption gets weaker (*E*_ads_ = −0.53 eV), and the energy barrier for dissociation is 0.41 eV. After CO adsorption, the COOH production requires a higher energy barrier of 0.85 eV. By comparing the LH mechanisms on the two models, it can be concluded that water is more favorable to be activated at the positively charged Cu cluster due to its Lewis acidity. Moreover, the energy barrier of water dissociation is significantly decreased with the assistance of the spilled-over oxygen atom by forming two hydroxyls. The exothermic reaction energy for water dissociation over Cu_14_/CeO_2_(111) indicates that the two hydroxyls may repel each other, which further facilitates carboxyl formation. Due to the electron donation of the CO 5σ orbital, the positively charged Cu atoms also favor the CO adsorption and stabilize the carboxyl group. After the removal of the two spilled-over oxygen, the Cu monolayer contains less positively charged Cu atoms (close to the Cu^0^ state), which accounts for the lower WGS reactivity. The oxygen vacancy can moderately adsorb water molecules, but the Cu atom involved in water dissociation neighbors the oxygen vacancy and is nearly neutral. The peripherical Cu^0^–V_O_–Ce^3+^ site is not the active site for water dissociation. The generated OH species can strongly interact with the neutral Cu atom, and therefore, COOH formation over Cu_14_–2V_O_ requires a higher energy barrier than the Cu_14_ monolayer.

Since the Mars–van Krevelen (MvK) mechanism on reducible oxides is also a possible catalytic mechanism for redox reactions, we also explored the WGS reaction *via* the MvK mechanism using Cu_14_/CeO_2_(111) (Fig. S10 and Table S3†), where the interfacial lattice oxygen vacancy affects the WGS reaction by capturing and activating water molecules, with the supported copper clusters providing the adsorption sites of CO molecules.^17^ It is found that the water adsorption energy on oxygen vacancies at the periphery of Cu_8_/CeO_2_(111) is −0.89 eV. The dissociation energy barrier is as high as 0.71 eV, compared to the LH mechanism on the model of Cu_14_/CeO_2_(111). The following CO adsorption energy is −1.19 eV, and the formation of COOH species overcomes a relatively high energy barrier of 0.73 eV. These energetic results indicate that the MvK mechanism is less favorable than the LH mechanism. This can be attributed to the strong interaction between the OH species and the oxygen vacancy that limits the formation of the COOH formation.

To directly represent the catalytic activity of the WGS reaction, we further performed micro-kinetic simulations to estimate the turn-over frequencies (TOF) and the conversions on the three catalyst models. Fig. 5b shows the comparison of WGS reaction rates as a function of temperature. Obviously, the existence of two spilled-over O atoms on the Cu monolayer of Cu_14_/CeO_2_(111) shows the highest reactivity of the WGS reaction *via* the LH mechanism at low temperature, while the WGS reaction *via* the MvK mechanism exhibits the lowest reactivity. It is further corroborated by the conversion of WGS reactions shown in Fig. 5c. The predicted lowest temperature of 100% conversion is 400, 460 and 538 K, respectively. We further simulated the infra-red spectra of water (H_2_O and D_2_O) molecules adsorbed on the supported Cu atoms or oxygen vacancies, as shown in Fig. S11.† The OD species adsorbed on Cu atoms in Cu_14_/CeO_2_(111) has an IR band at 2694 cm^−1^, while on Ce^3+^ (oxygen vacancies at the interface) the IR band position is blue-shifted to 2726 cm^−1^. Recently, Shen and co-workers observed the IR band of OD species adsorbed on copper-ceria catalysts at 2693 cm^−1^,^15^ and therefore, we deduce that the experimentally observed OD species is adsorbed on copper atoms not Ce^3+^ cations. These results suggest that the monolayer Cu cluster with lattice oxygen self-spilled over is highly reactive for the WGS reaction, and imply that the supported high oxidation-state Cu atoms in the Cu monolayer provide the active sites for WGS reactions, not the periphery of Cu–CeO_2_.

It is worth noting that CO oxidation may share some elementary steps with the WGS reaction.^47–49^ To explore whether under WGS reaction conditions the CO can reduce the O on the Cu cluster, the stability of the spilled-over O atoms above the Cu cluster was investigated. As shown in Fig. S12,† it takes 3.11 eV to remove the spilled-over O from the top of the Cu cluster, which is much harder than removing the O from the pure CeO_2_(111) surface (2.38 eV). In addition, removing O atoms at the periphery of Cu_14_/CeO_2_(111) is also hard (2.87 eV, Fig. S12†). That strong binding of the spilled-over O on the Cu cluster and the O atoms at the periphery makes their hard reduction by CO. As shown in Fig. S13,† the reactions between CO and these two types of O suffer from barriers as high as 0.83 and 1.29 eV, respectively. By contrast, the water dissociation and CO consumption along the WGS reaction in Fig. 5a is much more facile. In addition, further calculations have been done to investigate whether the dissociated H on the spilled-over O could form H_2_O together with the removal of O from the Cu site, but that process also requires a barrier as high as 1.17 eV (Fig. S14†), limiting its occurrence. Further micro-kinetic modeling also corroborates this point (Fig. S15†). All these calculations demonstrate the stability of spilled-over O on the Cu cluster and the favorable pathway in Fig. 5.

## 4 Conclusions

We have reported an unprecedented spillover of surface lattice oxygen from ceria support to Cu NCs. This hence causes a highly energetic preference of the monolayered configuration of the supported Cu nanocluster, compared to multilayered configurations. The Cu monolayer NCs can be highly dispersed with a promising stability and abundant positively charged Cu atoms on CeO_2_(111). The spilled-over oxygen and the adjacent positively charged Cu atoms in the Cu monolayer supported on CeO_2_ are further found to play a promotional role in WGS reactions.

In general, our work has proposed a new role of reducible oxides in heterogeneous catalysis that has not been realized previously. The surface lattice oxygen spillover from the oxide to its supported metal cluster may widely exist under realistic conditions, which may tune the catalyst structures and electronic properties, create new active sites, and ultimately improve the catalytic reactivity. However, we also note that Cu NCs may experience structural changes under WGS reaction conditions, and it is currently not clear how relevant it is to the corresponding activity. Further study on considering adsorption induced surface reconstruction would be significant in understanding the catalytic activity of Cu NCs. Similar studies can also be found in our previous reports on Au particles on reducible oxide supports.^50–52^

## Author contributions

Y. G. W. and Y. Q. S. conceived the idea and designed the calculation schemes. Y. Q. S. performed the DFT calculations; G. J. X. performed the AIMD simulations. All the authors contributed to writing the manuscript.

## Conflicts of interest

There are no conflicts to declare.

## Supplementary Material

SC-012-D1SC01201K-s001
