# Supplementary material for: Lattice oxygen self-spillover on reducible oxide supported metal cluster: the water–gas shift reaction on Cu/CeO2 catalyst
Source: Chem Sci. 2021 May 12;12(23):8260–7. doi: 10.1039/d1sc01201k (PMC8208302; doi:10.1039/d1sc01201k)
Supplement: SC-012-D1SC01201K-s001 [file SC-012-D1SC01201K-s001.pdf]

Electronic Supplementary Material (ESI) for Chemical Science.
This journal is © The Royal Society of Chemistry 2021

Electronic Supplementary Information

Lattice Oxygen Self-Spillover on Reducible Oxide Supported Metal Cluster: Water-Gas Shift Reaction on Cu/CeO_2 Catalyst

Ya-Qiong Su,[1,2,3,#] Guang-Jie Xia,[1,#] Yanyang Qin,[2] Shujiang Ding[2] and Yang-Gang Wang[1,*]

[1]Department of Chemistry and Guangdong Provincial Key Laboratory of Catalysis, Southern University of Science and Technology, Shenzhen, Guangdong 518055, China

[2]School of Chemistry, Xi'an Key Laboratory of Sustainable Energy Materials Chemistry, MOE Key Laboratory for Nonequilibrium Synthesis and Modulation of Condensed Matter, State Key Laboratory of Electrical Insulation and Power Equipment, Xi'an Jiaotong University, Xi'an 710049, China

[3]Laboratory of Inorganic Materials and Catalysis, Schuit Institute of Catalysis, Eindhoven University of Technology, P.O. Box 513, 5600 MB Eindhoven, The Netherlands

[#]These authors contributed equally: Ya-Qiong Su, Guang-Jie Xia.

* correspondence: wangyg@sustech.edu.cn

Fig. S1. Diffusion pathway and corresponding potential energy profile of single Cu atom on the $CeO_2(111)$ surface.

Fig. S2. DFT-optimized structures of several Cu_n clusters (n = 1-8, 14) on $CeO_2(111)$ and their correspondent adsorption energies.

Fig. S3. Relaxation process of Cu_6 on $CeO_2(111)$ using DFT.

Fig. S4. Structural extension of Cu clusters (Cu_7 to Cu_{14}) on $CeO_2(111)$.

Fig. S5. The spillover of ceria subsurface O (in green) into surface vacancies.

Fig. S6. The average radial distribution function (RDF, g(r)) of O atoms around the eight Cu atoms on CeO_2 in AIMD.

Fig. S7. Projected Density of states (pDOS) analysis of Cu single atom (a) and it anchored on perfect site (b) and oxygen vacancy (c) of the $CeO_2(111)$ surface.

Fig. S8. Intermediates of WGS over $Cu_{14}/CeO_2(111)$ with two O spillover (Cu14-a) via L-H (Langmuir-Hinshelwood) mechanism.

Fig. S9. Intermediates of WGS over $Cu_{14}/CeO_2(111)$ with two oxygen vacancies (VO) via L-H mechanism.

Fig. S10. Intermediates of WGS over $Cu_{14}/CeO_2(111)$ with two O spillover (Cu14-a) via MvK (Mars-van Krevelen) mechanism.

Fig. S11. Simulated IR spectra of (deuterated) water or produced OH after water dissociation on supported Cu_{14} and at the interfacial oxygen vacancy of $Cu_8/CeO_2(111)$, respectively.

Fig. S12. Removal of spilled-over O and the peripherical O atoms of $Cu_{14}/CeO_2(111)$.

Fig. S13. CO oxidation by the spilled-over O and peripherical O atoms of $Cu_{14}/CeO_2(111)$.

Fig. S14. The DFT-determined pathway of the dissociated H supported to form H_2O together with the removal of O from the Cu site.

Fig. S15. The Arrhenius plots of the rate of WGS reaction and the competitive H_2O formation from the dissociated H supported on $Cu_{14}/CeO_2(111)$ via LH mechanism.

Table S1. Bader charges of Cu atoms in $Cu/CeO_2(111)$ models.

Table S2. Energies data of WGS by the selected models of Cu/CeO_2.

Supplementary Computational Details

Structure Optimization by GA-DFT. GA-DFT calculations have been conducted to find the global minimum structures of CeO_2(111)-supported Cu_8 and Cu_{14} NCs at the level of PBE functional. The first step for GA run is to generate the initial population (12 random starting candidates used here) randomly. All the starting candidates should be reasonable with the bond distances in the region of 2 Å ~ 3Å avoiding redundant calculations. Then, DFT calculations have been performed to calculate the energy of each structure. During the GA-DFT calculations, the relative energies and sorted distance matrices are used to judge whether the two candidates have the same structures. The calculated energies of the structures are used in the assigning of the fitness and the structures with lower energy have higher possibility to be maintained in the population. The fitness function of ith candidate (Fi) can be calculated as below[1] :

$$F_i = ½[1 - \tanh(2\rho_i - 1)] \text{ with } \rho_i = (E_i - E_{min})/(E_{max} - E_{min}) \quad (1)$$

E_{max} and E_{min} is the maximum and minimum energy of any structure in the population.

After assigning the fitness, we generate new population by crossover, as introduced by Deaven and Ho,[2-3] and mutation operations which are the most important and key operators in GA. For crossover operation, we first choose two random cutting planes (m1 and m2) and position it in the common center of father structure C1 and structure C2, respectively. By generating new structure, we select all atoms from C1 which are to the left of the plane m1 and all atoms from the structure C2 which are to the right of the plane m2. If the total number of new structures (N_n) is larger than N, we remove the N_n-N atoms which are farthest away of the new structure. However, if $N_n < N$, we randomly chose N-Nn atoms from C2 which were not previously included. The generated new structure should have reasonable bond distances and be different with other structures in the population.

For the mutation operation in GA, the rattle and twist operation are considered. Firstly, 40% atoms in transition metal clusters are allowed to move a random distance in a random direction with the perturbations between -0.6 Å and 0.6 Å. Then, the adsorbed cluster can be rotated a random angle ranging from 1° – 180° with respect to the surface normal. After the mutation operation, we have to confirm the new generated structures are reasonable with suitable bonds distances. The random number is generated by Mersenne Twister random number generator.

The above steps are cycled to optimize and generate more and more populations. More than 500 structures have been calculated and no new structure are obtained after 100 cycles.

Micro-kinetic Modeling. The calculated activation energies are applied to compute the forward and backward rate constants for WGS reactions. For surface reactions, the rate constants for the forward and backward elementary reaction were determined by the Eyring equation[4]:

$$k = \frac{k_b T}{h}\frac{Q^{TS}}{Q} e^{-\frac{Ea}{k_b T}} \quad (2),$$

where k is the reaction rate constant in s^{-1}; k_b, T, h and E_a are the Boltzmann constant, temperature, Planck's constant and the activation barrier, respectively. Q^{TS} and Q refer the partition functions of the transition and ground states, respectively. As an approximation, the pre-factor $A = \frac{k_b T}{h}\frac{Q^{TS}}{Q}$ is set to 10^{13} s^{-1} for all the elementary surface reactions.

For non-activated molecular adsorption, the rate of adsorption is determined by the rate of surface impingement of gas-phase molecules. The flux of incident molecules is given by Hertz-Knudsen equation[5]:

$$F = \frac{P}{\sqrt{2\pi m k_b T}} \quad (3).$$

Therefore, the molecular adsorption rate constant can be written as:

$$k_{ads} = \frac{PA'}{\sqrt{2\pi m k_b T}} S \quad (4),$$

where P is the partial pressure of the adsorbate in the gas phase, A' is the surface area of the adsorption site, m *is* the mass of the adsorbate, and S is the sticking coefficient.

For the desorption process, it is assumed that there are three rotational degrees of freedom and two translational degrees of freedom in the transition state. Accordingly, the rate of desorption is given by

$$k_{des} = \frac{k_b T^3}{h^3}\frac{A'(2\pi k_b)}{\sigma\theta_{rot}} e^{-\frac{E_{des}}{k_b T}} \quad (5),$$

where σ and θ are the symmetry number and the characteristic temperature for rotation of gaseous molecules, respectively. E_{des} is the desorption energy of gaseous molecules.

The approach to microkinetic simulations has been presented in detail elsewhere.[6-7] Differential equations for all the surface reaction intermediates were constructed using the rate constants and

the set of elementary reaction steps. For each of the *M* components in the kinetic network, a single differential equation is obtained in the form as below:

$$r_i = \sum_{j=1}^{N}\left(k_j v_i^j \prod_{k=1}^{M} c_k^{v_k^j}\right) \quad (6),$$

in this equation, k_j is the elementary reaction rate constant (see Equation 2), v_i^j is the stoichiometric coefficient of component *i* in elementary reaction step *k* and c_k is the concentration of component *k* on the catalytic surface.

The WGS reaction rate is calculated by the in-house developed MKMCXX program.[6-8] Steady-state coverages were calculated by integrating the ordinary differential equations in time until the changes in the surface coverages were very small. Because chemical systems typically give rise to stiff sets of ordinary deferential equations (ODEs), we have used the backward differentiation formula method for the time integration.[7] The rates of the individual elementary reaction steps can be obtained based on the calculated steady-state surface coverages. In our simulations, the gas phase contained a mixture of CO and O_2 in 2:1 molar ratio at a total pressure of 1 atm.

The elementary reaction steps that contribute to the rate control over the overall reaction can be determined by degree of rate control (DRC) concept introduced by Campbell et al.[9-11] For elementary step *i*, the degree of rate control $X_{RC,i}$ can be defined as

$$X_{\mathrm{RC},i} = \frac{k_i}{r}\left(\frac{\partial r}{\partial k_i}\right)_{k_{j\neq i},K_i} = \left(\frac{\partial \ln r}{\partial \ln k_i}\right)_{k_{j\neq i},K_i} \quad (7),$$

where k_i, K_i and *r* are the rate constants, the equilibrium constant for step *i* and the reaction rate, respectively.

Hubbard terms in DFT+*U* approach. In this work, the DFT calculations were done by VASP package, and the typical Hubbard parameter of Ce 4f electrons (U_{eff} = 4.5 eV) was used, which was calculated self-consistently by Fabris et al.[12,13] and agrees well with the report by Castleton et al.[14]. Yet, due to the high computational cost, the AIMD simulations in VASP package were quite slow. Hence, the CP2k package was used to do the AIMD simulations because of its faster speed. In VASP package, the pure plane waves are used as the basis set to solve the Kohn-Sham equations, but in CP2k, essentially the Gaussian type basis sets are used (although those Gaussian orbitals should also be expanded to plane waves in CP2k calculations). Due to the differences, the Hubbard

parameter in VASP cannot be directly applied to CP2k. Several tests were done. We found by using the Gaussians and auxiliary plane waves (GPW) double zeta Gaussian basis set in CP2k, when no Hubbard term of Ce 4f electrons (U_{eff} = 0 eV) was applied, the work function of our system was around similar to that calculated by VASP. More importantly, by this setting, the O spillover energy of monolayered Cu_8 on $CeO_2(111)$ in CP2k is exothermic by around 1 eV, which well agrees with that value in VASP shown in Fig. 2a. These tests could in some sense ensure our calculations at the same footage.

AIMD Simulation Temperature. In the manuscript, the AIMD simulation was done at 773K (500℃). That is the typical temperature of the calcination during the preparation of Cu/CeO_2 catalyst in experiments. (For example, the Cu/CeO_2 catalysts were calcinated at 400℃ in [15]; 300−450℃ in [16]; 500℃ in [17]; 650℃ in [18]) Although the low-T WGS catalysts typically work below 600K, the AIMD simulation at the calcination temperature could reveal the state of the as-prepared catalyst, which can then be used to catalyze the low-T WGS reaction.

In our system, as shown in Fig. 2, the O spillovers in Cu_8/CeO_2 and Cu_{14}/CeO_2 are both quite exothermic with the barriers of 0.53 eV and 0.69 eV, respectively. Applying the Arrhenius equation ($r = Ae^{-\frac{E_a}{RT}}$, simply estimate the pre-exponential factor A at the scale of 10^{12} s^{-1}), these O spillover barriers correspond to the estimated rates of 3.50×10^8 s^{-1} and 3.17×10^7 s^{-1} respectively at 773 K. Even at room temperature (300 K), they still have the rate of 1.25×10^3 s^{-1} and 2.56 s^{-1} respectively. These reaction rates, together with their exothermic reaction energies, suggest the O spillover could also take place at the temperatures of low-T WGS reactions.

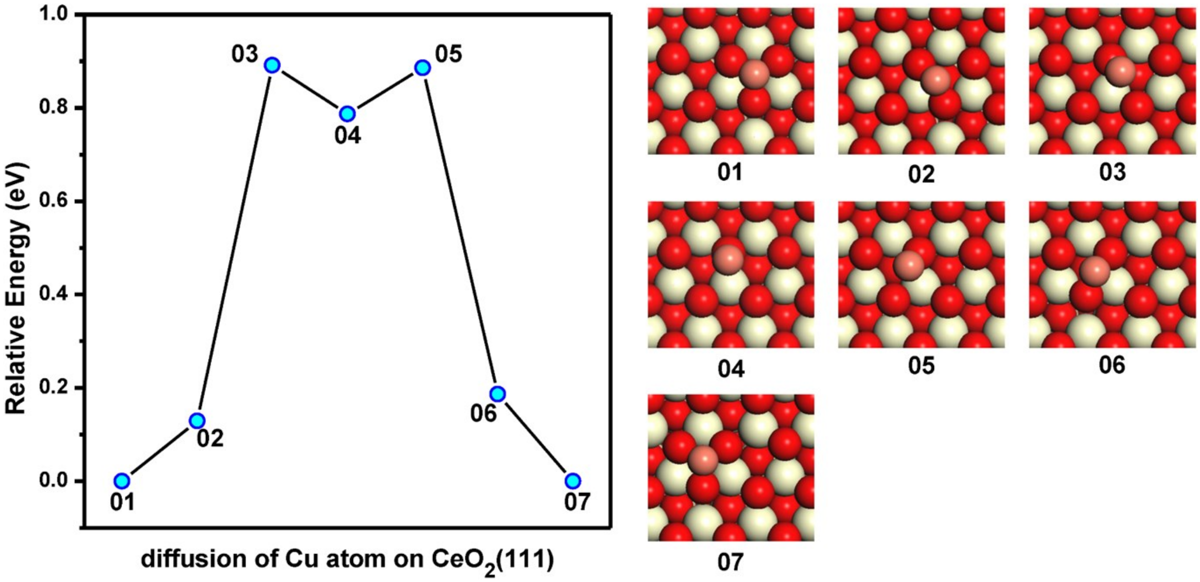

Fig. S1. Diffusion pathway and corresponding potential energy profile of single Cu atom on the CeO_2(111) surface. Color code: red, O; pink, Cu; white, Ce.

The pathway was determined by CI-NEB method. Cu atom prefers to be bound to three surface lattice O atoms, and the adsorption energy is -2.90 eV. The required energy barrier of Cu atom diffusion on CeO_2(111) is 0.89 eV. According to Arrhenius equation $r = \frac{k_B T}{h} e^{-E_a/RT}$, the Cu diffusion rate constant is estimated to be 2.5×10^7 s^{-1} at the typical reaction temperature of 773 K in experiment, which indicates single Cu atoms are extremely unstable and make for sintering via ripening to form large clusters or particles on CeO_2(111) under typical synthesis temperatures of Cu/CeO_2 catalysts.

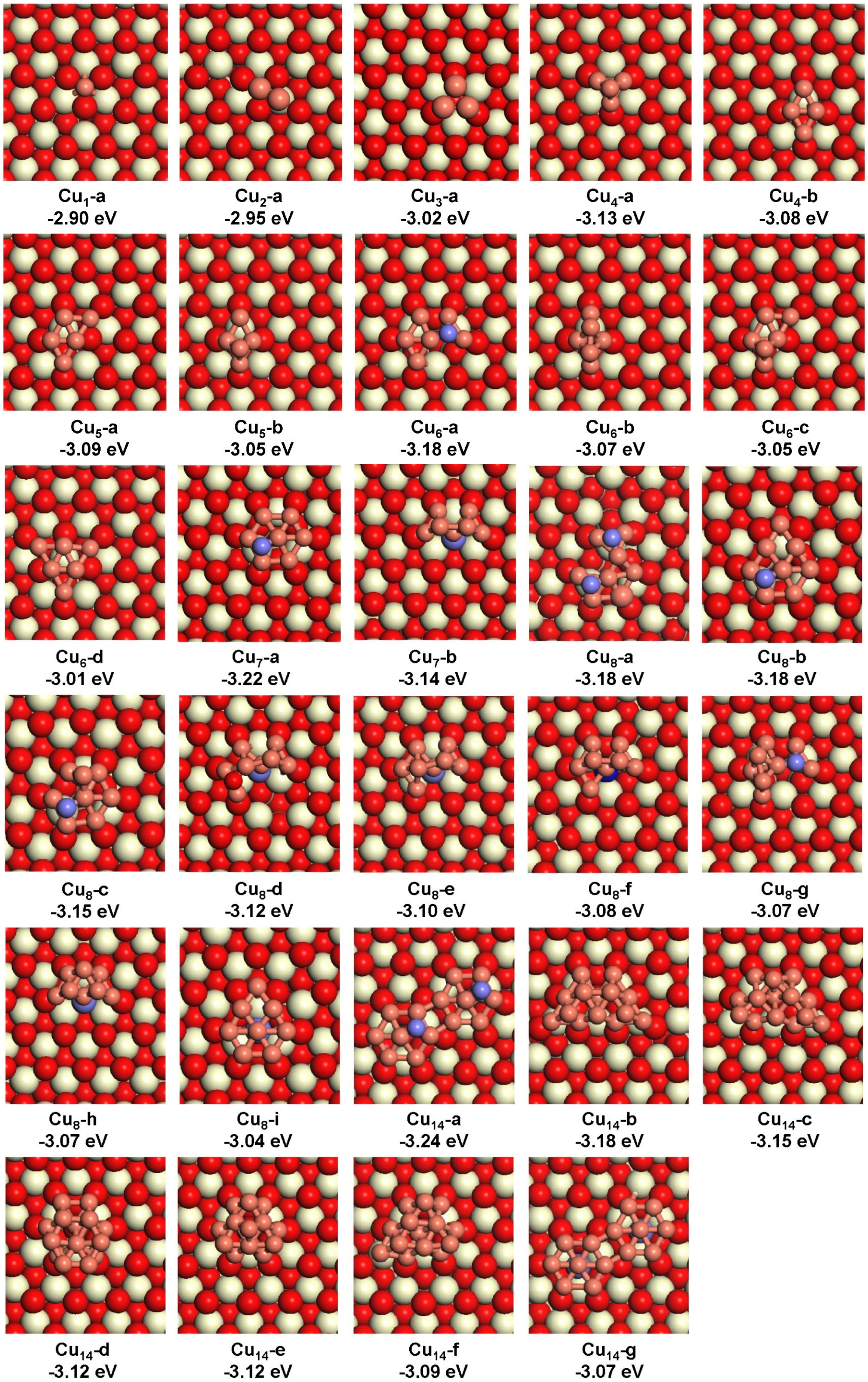

Fig. S2. DFT-optimized structures of several Cu_n clusters (n = 1-8, 14) on $CeO_2(111)$ and their correspondent adsorption energies. Color code: red, O; pink, Cu; white, Ce; blue, spilled O.

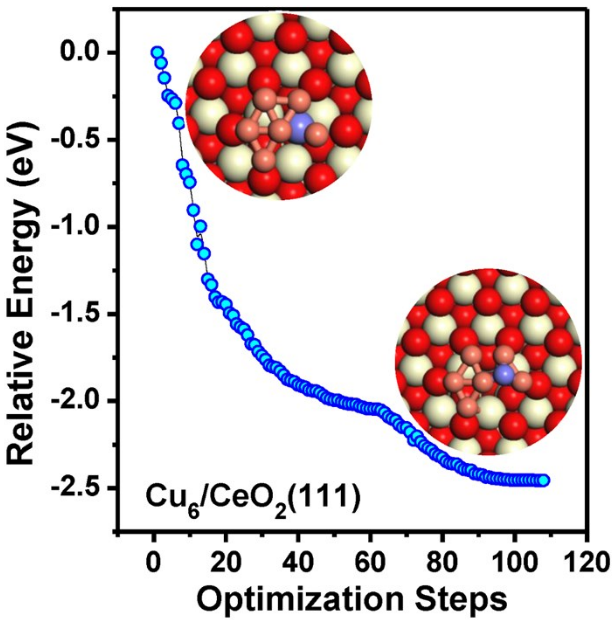

Fig. S3. Relaxation process of Cu_6 on $CeO_2(111)$ using DFT. The initial configuration is organized by adding one Cu atom to the planar $Cu_5/CeO_2(111)$ and then optimized using DFT. Finally, one lattice O atom (blue ball) was spontaneously spilled over on the planar $Cu_6/CeO_2(111)$. Colour code: red, O; pink, Cu; white, Ce; blue, spilled O.

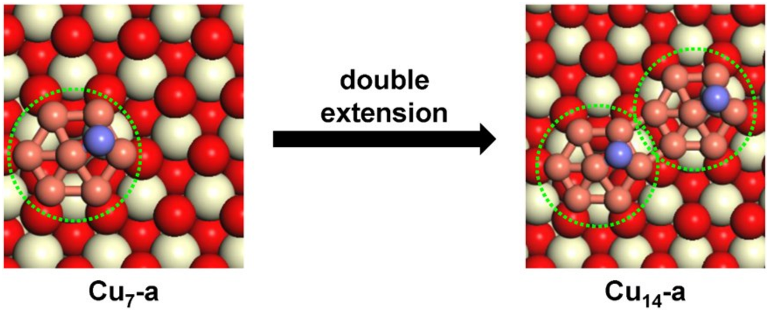

Fig. S4. Structural extension of Cu clusters (Cu_7 to Cu_{14}) on $CeO_2(111)$. Double extension of the monolayered Cu cluster with O spillover in Cu_7-a can produce the initial configuration of the monolayered Cu_{14}-a which indeed contains two Cu_7 template shown in green dashed circles. After construction, the configurations have been further optimized, and their stabilities are also confirmed by global search.

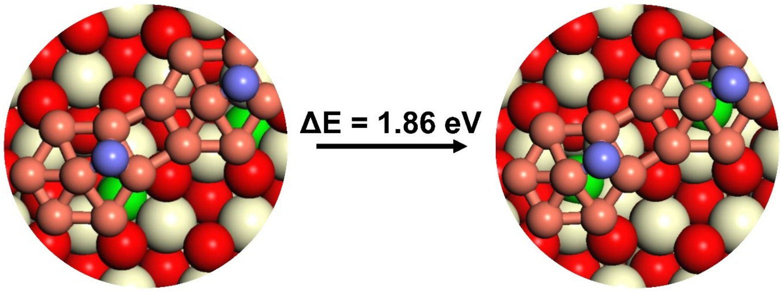

Fig. S5. The spillover of ceria subsurface O (in green) into surface vacancies.

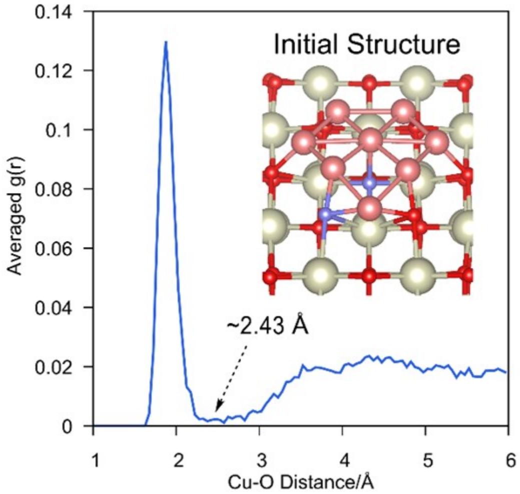

Fig. S6. The average radial distribution function (RDF, g(r)) of O atoms around the eight Cu atoms on CeO_2 in AIMD. The curve is averaged by the eight curves of each Cu atom.

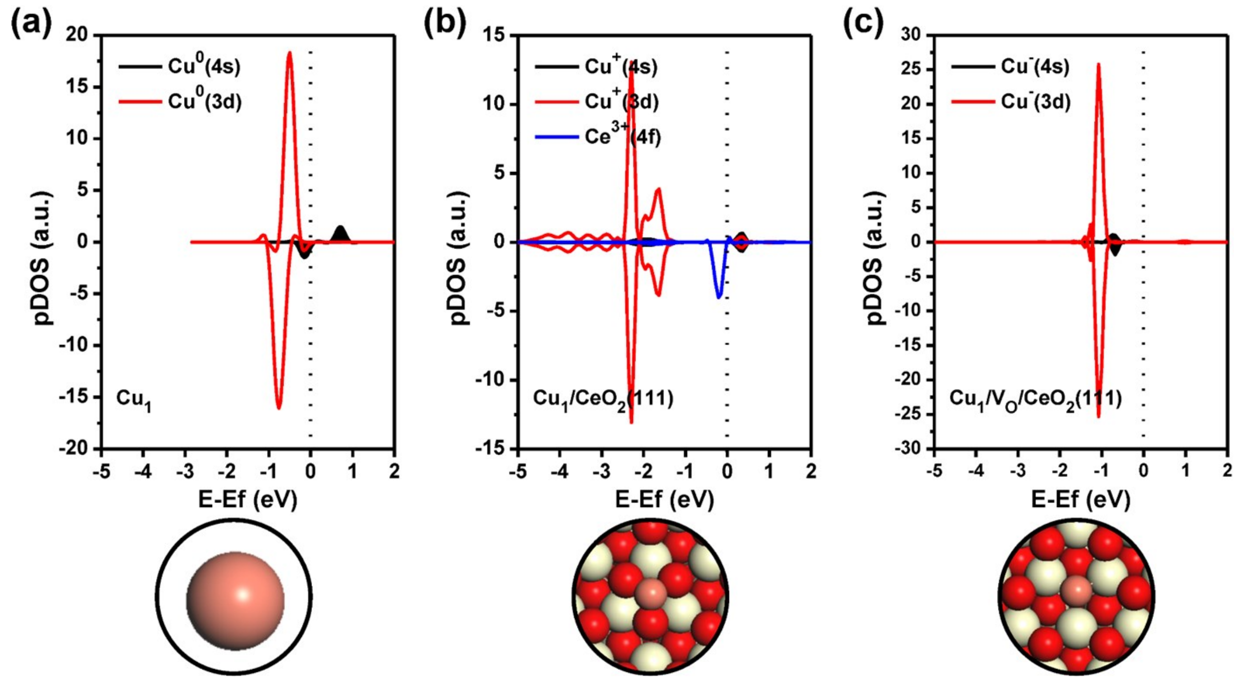

Fig. S7. Projected Density of states (pDOS) analysis of Cu single atom (a) and it anchored on perfect site (b) and oxygen vacancy (c) of the $CeO_2(111)$ surface.

For neutral free Cu atom, there is an asymmetric occupied peak corresponding to the Cu 4s state. For Cu^+ in $Cu_1/CeO_2(111)$, there are extremely weak symmetric peaks above Fermi level and in turn one Ce^{3+} is formed, while for Cu^- in $Cu_1/V_O/CeO_2(111)$, those are below Fermi level. The adsorption of Cu atoms on oxygen vacancy can cause electron transfer from Ce^{3+} into Cu atoms and resultingly the oxidation state of Cu atoms is decreased.

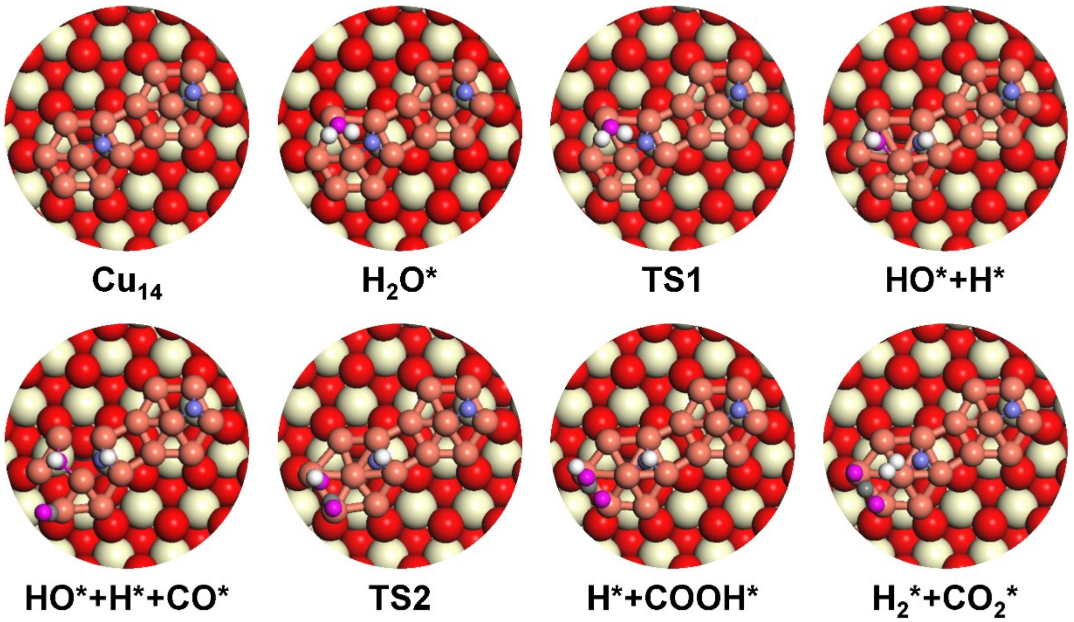

Fig. S8. Intermediates of WGS over $Cu_{14}/CeO_2(111)$ with two O spillover (Cu14-a) via L-H (Langmuir-Hinshelwood) mechanism.

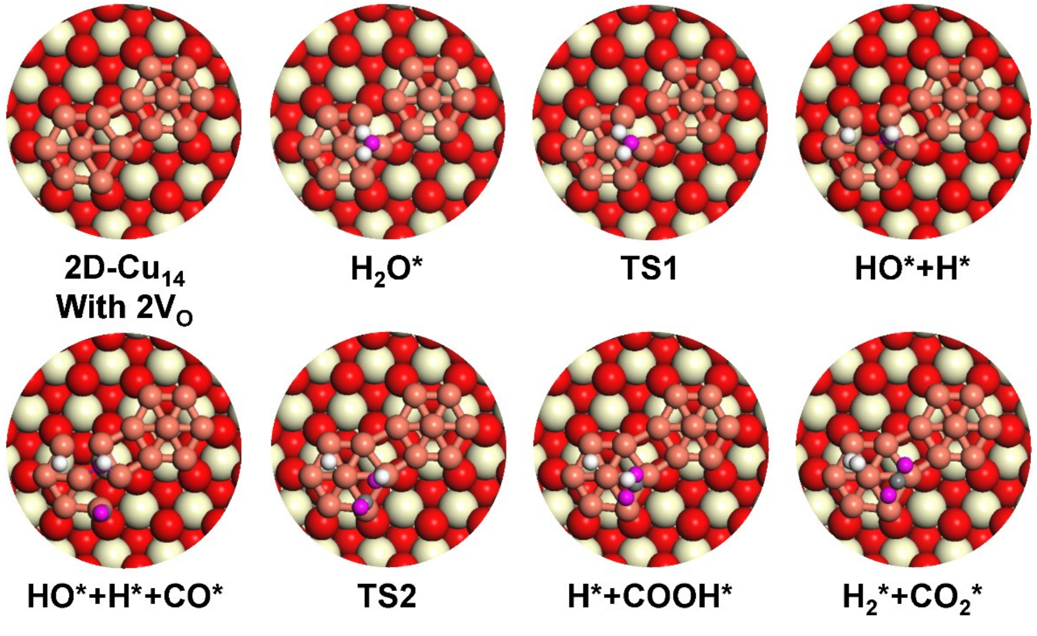

Fig. S9. Intermediates of WGS over $Cu_{14}/CeO_2(111)$ with two oxygen vacancies (VO) via L-H mechanism.

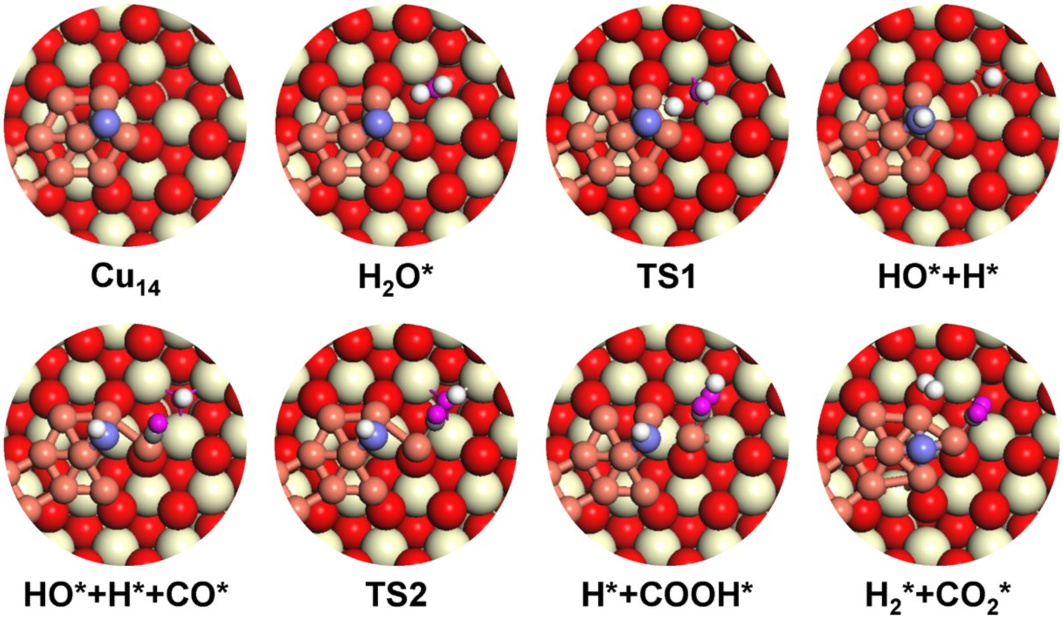

Fig. S10. Intermediates of WGS over $Cu_{14}/CeO_2(111)$ with two O spillover (Cu14-a) via MvK (Mars-van Krevelen) mechanism.

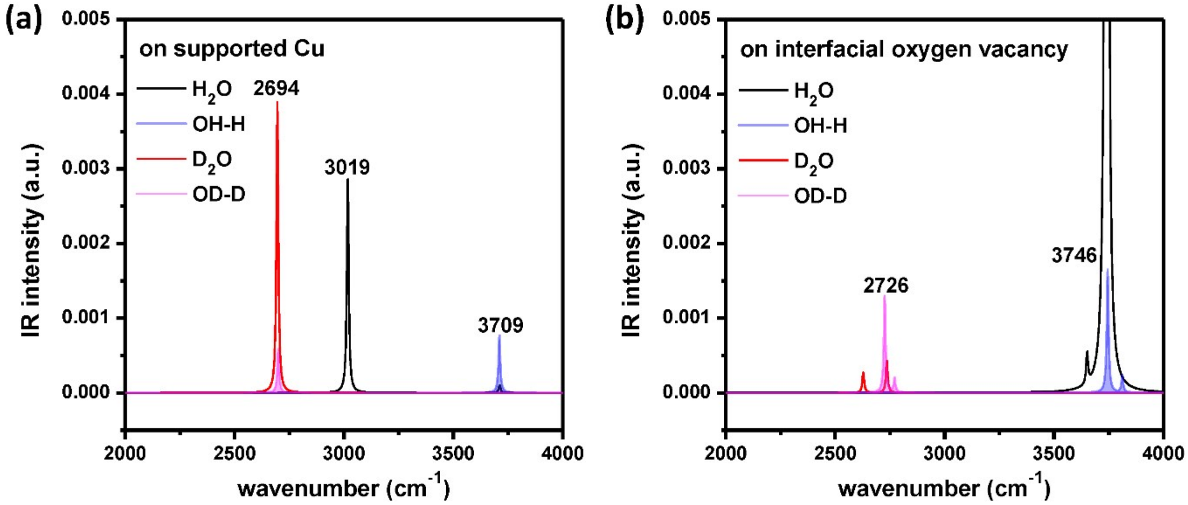

Fig. S11. Simulated IR spectra of (deuterated) water or produced OH after water dissociation on supported Cu_{14} and at the interfacial oxygen vacancy of $Cu_8/CeO_2(111)$, respectively. The scaling factor is 1.0099, which is selected referring to the stretching vibrational mode of CO in gas phase (theoretical 2122 cm^{-1} vs. experimental 2143 cm^{-1}).

The OD species adsorbed on Cu atoms in $Cu_{14}/CeO_2(111)$ has an IR band at 2694 cm^{-1}, while on Ce^{3+} (oxygen vacancy at the interface) the IR band position is blue-shifted to 2726 cm^{-1}. Recently, Shen and co-workers observed IR band of OD species adsorbed on copper-ceria catalysts at 2693 cm^{-1}, and therefore, we suggested that the experimental observed OD species is adsorbed on copper atom not Ce^{3+} cations. It further supported that WGS reactions take place via LH mechanism, not MvK mechanism.

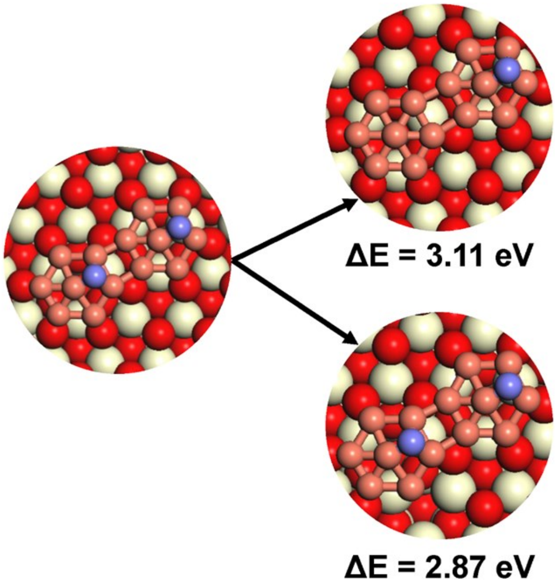

Fig. S12. Removal of spilled-over O and the peripherical O atoms of $Cu_{14}/CeO_2(111)$.

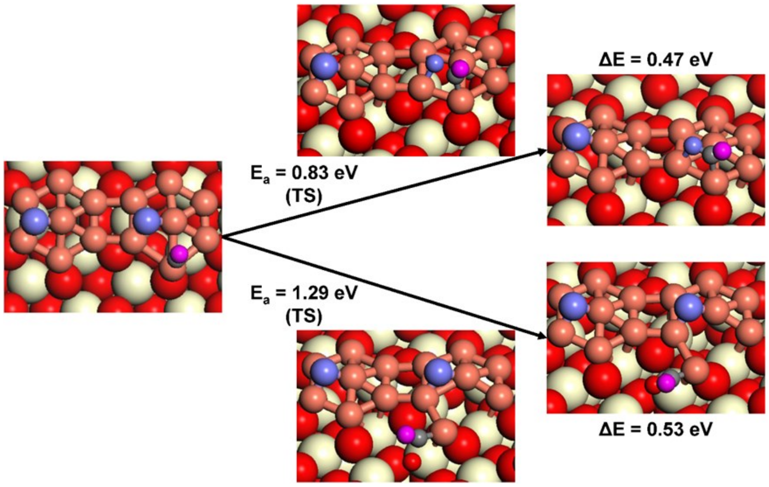

Fig. S13. CO oxidation by the spilled-over O and peripherical O atoms of $Cu_{14}/CeO_2(111)$.

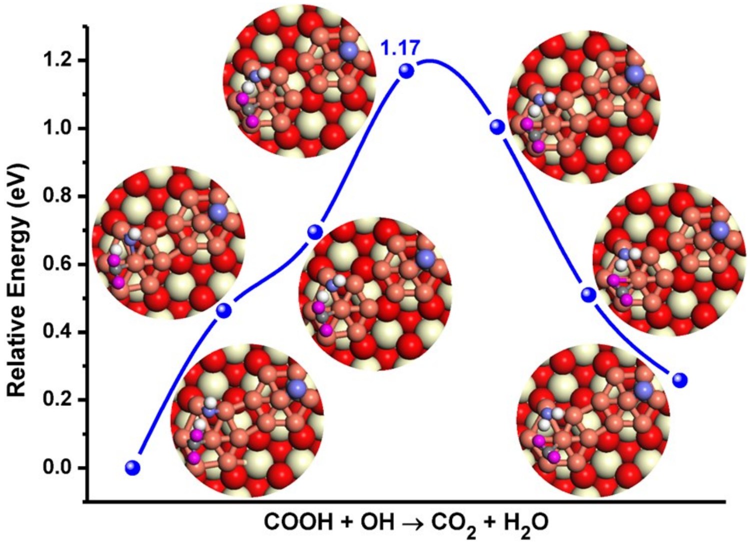

Fig. S14. The DFT-determined pathway of the dissociated H supported to form H_2O together with the removal of O from the Cu site.

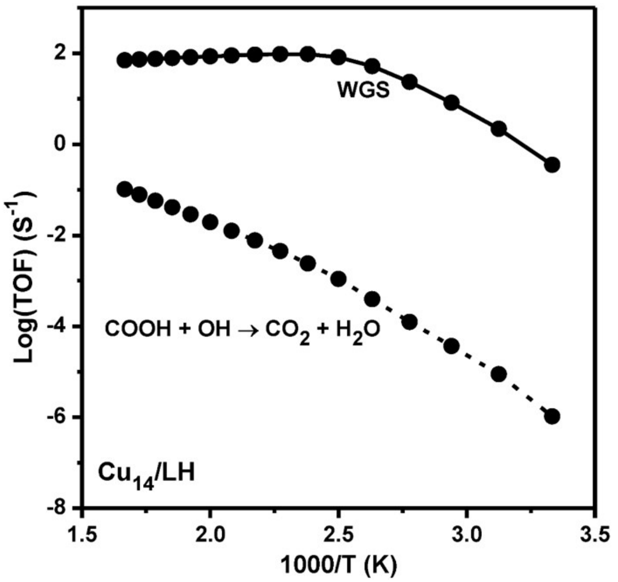

Fig. S15. The Arrhenius plots of the rate of WGS reaction and the competitive H_2O formation from the dissociated H supported on $Cu_{14}/CeO_2(111)$ via LH mechanism.

Table S1. Bader charges of Cu atoms in $Cu/CeO_2(111)$ models.

Species	Bader charge of Cu (+e)	number of Ce^{3+}
Cu_2O (Cu^+)	0.54	-
CuO (Cu^{2+})	1.00	-
Cu_1/CeO_2 (Cu1-a)	0.54	1
Cu_2/CeO_2 (Cu2-a)	0.44, 0.41	2
Cu_3/CeO_2 (Cu3-a)	0.18, 0.19, 0.15	1
Cu_4/CeO_2 (Cu4-a)	0.25, 0.25, 0.27, 0.10	2
Cu_5/CeO_2 (Cu5-a)	0.10-0.20 (2 atoms), 0.20-0.25 (2 atoms), 0.44	3
Cu_6/CeO_2 (Cu6-a)	0.10-0.20 (3 atoms), 0.20-0.25 (1 atoms), 0.49 (2 atoms)	4
Cu_7/CeO_2 (Cu7-a)	0.10-0.20 (2 atoms), 0.20-0.25 (3 atoms), 0.49 (2 atoms)	5
Cu_8/CeO_2 (Cu8-a)	0.10-0.20 (2 atoms), 0.20-0.25 (2 atoms), 0.45 (4 atoms)	6
Cu_{14}/CeO_2 (Cu14-a)	0.10-0.20 (4 atoms), 0.20-0.25 (6 atoms), 0.45-0.50 (4 atoms)	10
Cu_{14}-$2V_O/CeO_2$	-0.11 (2 atoms), 0.10-0.20 (11 atoms), 0.20-0.25 (1 atom)	9

Table S2. Energies data of WGS by the selected models of Cu/CeO_2.

supports	M	E_{ads} (H_2O)	E_a (TS1)	ΔE (HO*+H*)	E_{ads} (CO)	E_a (TS2)	ΔE (COOH*)	ΔE (CO_2+H_2)
Cu_{14}	LH	-0.95	0.03	-0.36	-1.09	0.46	0.06	1.66
Cu_{14}-$2V_O$	LH	-0.53	0.41	-0.86	-1.06	0.85	0.27	1.50
Cu_{14}	MvK	-0.96	0.85	-0.63	-1.11	0.80	0.65	1.37

References:

(1) Vilhelmsen, L. B.; Hammer, B. A genetic algorithm for first principles global structure optimization of supported nano structures. *J. Chem. Phys.* **2014,** *141*, 044711.
(2) Deaven, D. M.; Ho, K.-M., Molecular geometry optimization with a genetic algorithm. *Phys. Rev. Lett.* **1995,** *75*, 288.
(3) Daven, D.; Tit, N.; Morris, J.; Ho, K. Structural optimization of Lennard-Jones clusters by a genetic algorithm. *Chem. Phys. Lett.* **1996,** *256*, 195-200.
(4) Eyring, H. The activated complex in chemical reactions. *J. Chem. Phys.* **1935,** *3*, 107-115.
(5) Nitoń, P.; Żywociński, A.; Fiałkowski, M.; Hołyst, R. A "nano-windmill" driven by a flux of water vapour: a comparison to the rotating ATPase. *Nanoscale* **2013,** *5*, 9732-9738.
(6) Filot, I. A. W.; van Santen, R. A.; Hensen, E. J. M. The optimally performing Fischer–Tropsch catalyst. *Angew. Chem.* **2014,** *126*, 12960-12964.
(7) Filot, I. A. W.; Broos, R. J.; van Rijn, J. P.; van Heugten, G. J.; van Santen, R. A.; Hensen, E. J. M. First-principles-based microkinetics simulations of synthesis gas conversion on a stepped rhodium surface. *ACS Catal.* **2015,** *5*, 5453-5467.
(8) http://www.mkmcxx.nl/, http://www.mkmcxx.nl/
(9) Campbell, C. T. Future directions and industrial perspectives micro-and macro-kinetics: their relationship in heterogeneous catalysis. *Topics in Catal.* **1994,** *1*, 353-366.
(10) Campbell, C. T. Finding the rate-determining step in a mechanism: Comparing DeDonder relations with the "degree of rate control". *J. Catal.* **2001,** *204*, 520-524.
(11) Stegelmann, C.; Andreasen, A.; Campbell, C. T. Degree of rate control: How much the energies of intermediates and transition states control rates. *J. Am. Chem. Soc.* **2009,** *131*, 8077-8082.
(12) Fabris, S.; De Gironcoli, S.; Baroni, S.; Vicario, G.; Balducci, G. Taming Multiple Valency with Density Functionals: A Case Study of Defective Ceria. *Phys. Rev. B - Condens. Matter Mater. Phys.* **2005**, *71* (4), 041102.
(13) Fabris, S.; De Gironcoli, S.; Baroni, S.; Vicario, G.; Balducci, G. Reply to "Comment on 'Taming Multiple Valency with Density Functionals: A Case Study of Defective Ceria.'" *Phys. Rev. B - Condens. Matter Mater. Phys.* **2005**, *72* (23), 237102.
(14) Castleton, C. W. M.; Kullgren, J.; Hermansson, K. Tuning LDA+U for Electron Localization and Structure at Oxygen Vacancies in Ceria. *J. Chem. Phys.* **2007**, *127* (24), 244704.
(15) Ahn, S. Y.; Na, H. S.; Jeon, K. W.; Lee, Y. L.; Kim, K. J.; Shim, J. O.; Roh, H. S. Effect of Cu/CeO2 Catalyst Preparation Methods on Their Characteristics for Low Temperature Water−gas Shift Reaction: A Detailed Study. *Catal. Today* **2020**, *352*, 166–174.

(16) Chen, C.; Zhan, Y.; Zhou, J.; Li, D.; Zhang, Y.; Lin, X.; Jiang, L.; Zheng, Q. Cu/CeO $_2$ Catalyst for Water-Gas Shift Reaction: Effect of CeO $_2$ Pretreatment. *ChemPhysChem* **2018**, *19* (12), 1448–1455.
(17) Zhang, Y.; Liang, L.; Chen, Z.; Wen, J.; Zhong, W.; Zou, S.; Fu, M.; Chen, L.; Ye, D. Highly Efficient Cu/CeO2-Hollow Nanospheres Catalyst for the Reverse Water-Gas Shift Reaction: Investigation on the Role of Oxygen Vacancies through in Situ UV-Raman and DRIFTS. *Appl. Surf. Sci.* **2020**, *516*, 146035.
(18) Qi, X.; Flytzani-Stephanopoulos, M. Activity and Stability of Cu-CeO2 Catalysts in High-Temperature Water-Gas Shift for Fuel-Cell Applications. *Ind. Eng. Chem. Res.* **2004**, *43* (12), 3055–3062.
